# Long term anticoagulation for Catheter-Related deep vein thrombosis of the upper extremities in women with cancer: retrospective analysis of effectiveness and safety outcomes

**DOI:** 10.1007/s11239-025-03182-3

**Published:** 2025-09-26

**Authors:** Chiara Cavallaro, Paolo Santini, Laura Leoni, Carolina Mosoni, Silvia D’Ambrosio, Francesco Mancinetti, Nicola Coletta, Michela Iorio, Angelo Porfidia, Alessandro D’Errico, Rosa Talerico, Roberto Pola

**Affiliations:** 1 Thrombosis Unit, Department of Aging, Orthopedic, and Rheumatologic Sciences, Fondazione Policlinico Universitario A. Gemelli IRCCS, Rome, Italy; 2 Università Cattolica del Sacro Cuore, Rome, Italy; 3 Vita-Salute University, San Raffaele Hospital IRCCS, Milan, Italy

**Keywords:** Central venous catheter, Upper extremity deep vein thrombosis, Anticoagulation, Venous thromboembolism, Active cancer, Women

## Abstract

Catheter-related upper extremity deep vein thrombosis (CRT-UEDVT) is a possible complication in patients with cancer carrying a central venous catheter. Anticoagulation is the primary treatment, but optimal duration is unclear. This study evaluated effectiveness and safety of different lengths of anticoagulation in women with cancer and CRT-UEDVT. We conducted a retrospective analysis on women ≥ 18 years-old, who had active cancer and had received anticoagulant treatment for CRT-UEDVT. Effectiveness was assessed in terms of VTE recurrence and thrombosis recanalization. Safety was determined by assessing major bleedings (MB) and clinically relevant non-major bleedings (CRNMB) during treatment. A total of 113 women where included. All of them had completed at least 3 months of anticoagulant therapy, while 106 and 97 had completed 6 and 12 months of anticoagulant therapy, respectively. The median follow-up was 568.5 days (IQR 300–910). Patients primarily presented with ovarian, breast, and endometrial cancers. Anticoagulant therapy was mainly parenteral during the initial 3 months and between 3 and 6 months, shifting predominantly to direct oral anticoagulants during months 6–12. The annual VTE recurrence rate was 0.5%. The annual rate of MB and CRNMB was 1.9%. Complete thrombosis recanalization was achieved in 52.0%, 69.1%, and 87.3% of patients at 3, 6, and 12 months, respectively. Our study provides interesting insights into the management and clinical outcomes of women with cancer and CRT-UEDVT. Prospective studies are needed to fully understand advantages and disadvantages of different lengths of anticoagulation in this set of patients.

**Graphical abstract:**

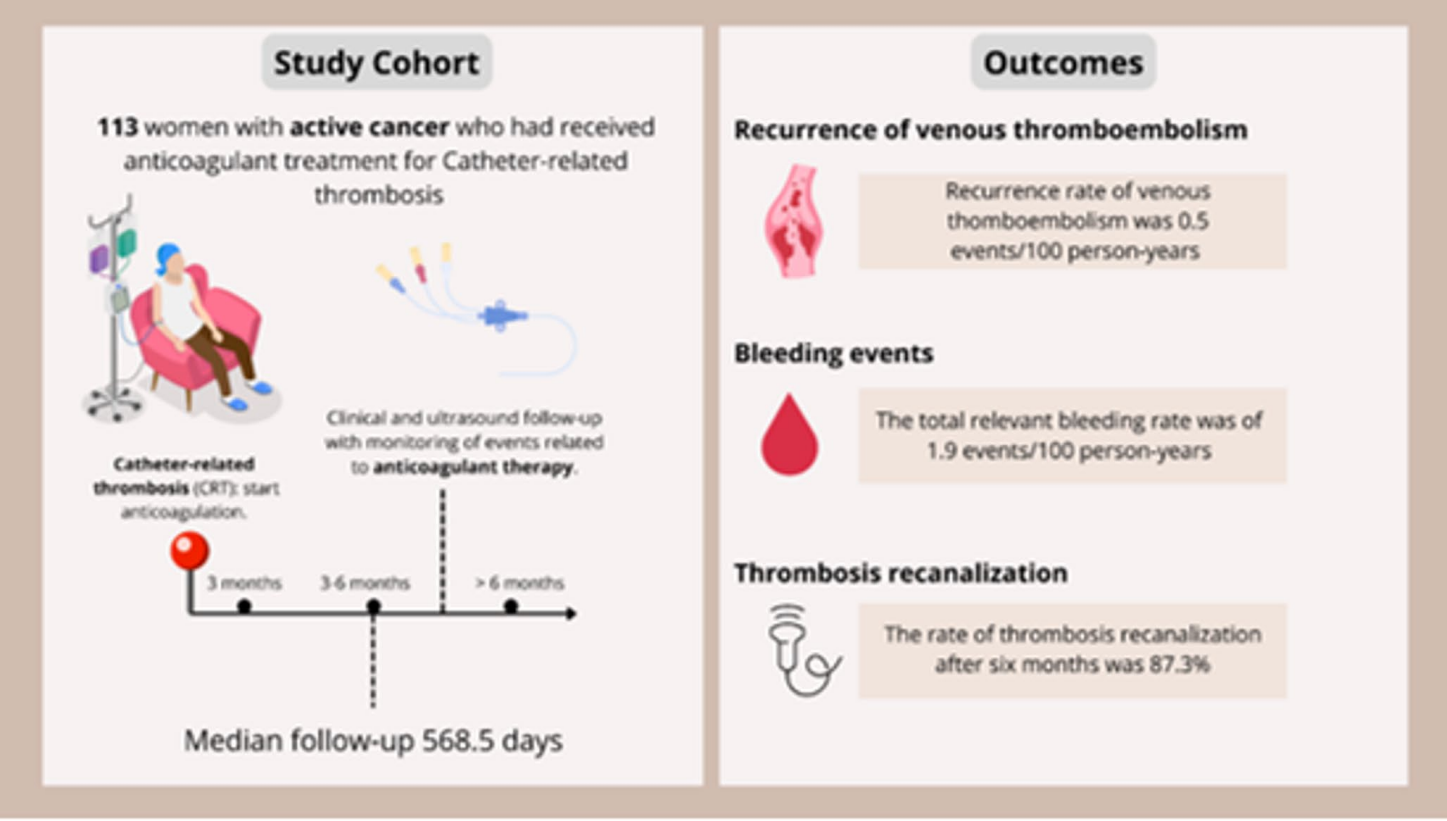

**Supplementary Information:**

The online version contains supplementary material available at 10.1007/s11239-025-03182-3.

## Introduction

Central venous catheters (CVCs) are routinely utilized for the administration of medications, fluids, dialysis and parenteral nutrition. These devices are classified based on their design, anticipated duration of use, and insertion site. Non-tunneled short-term catheters are typically employed in emergency or acute inpatient settings, whereas long-term central venous access devices (CVADs), including Hickman catheters, Port-a-Cath, and Peripherally Inserted Central Catheters (PICC), are preferentially used for the management of outpatients with an oncological disease and can remain in place for extended periods, ranging from months to years [1]. The use of CVCs is associated with possible complications, including catheter-related thrombosis (CRT) [2]. The reported rate of CRT at the level of the deep veins of the upper extremities is 2.7 per 1,000 catheter-days [95% Confidence Interval (95% CI) 1.0-6.2) [2, 3]. According to the literature, CRT represents 70–80% of all upper extremity deep vein thromboses (UEDVT) and 10% of all cases of venous thromboembolism (VTE) [4]. Since CRT-UEDVT may be asymptomatic, the true incidence of this complication might be higher than reported. The pathophysiology of CRT-UEDVT is multifactorial. In patients with cancer, systemic factors associated with the disease, local factors linked to vascular injury during catheter insertion, and patient-related factors such as previous history of VTE and thrombophilic conditions, may contribute [5]. The type of catheter also plays a significant role, with PICC being 2–3 times more likely to induce thrombosis compared to central catheters [6]. Furthermore, characteristics such as catheter size, number of lumens, improper tip placement, and technical complications, including difficulties during insertion and multiple attempts, are consistently associated with increased incidence of CRT [7, 8].

The optimal therapeutic management of CRT-UEDVT is still debated, since randomized clinical trials on this disease do not exist and data are derived only from a limited number of small-scale observational studies. Therefore, optimal type, intensity, and duration of anticoagulation in this clinical condition have not yet been established. The goal of treatment is to improve symptoms of the acute thrombosis, restore CVC patency, favour thrombosis recanalization, and prevent recurrent thromboembolic events. Low-molecular-weight heparins (LMWHs) are typically advised over unfractionated heparin due to their more favourable safety profile and ease of use [9]. Studies comparing direct oral anticoagulants (DOACs) with LMWHs suggest that DOACs provide similar results in terms of recurrence, bleeding, and mortality [10–12]. Duration of treatment is controversial, but current guidelines recommend a minimum anticoagulation of 3 months, and to continue if the catheter is in place [13]. However, treatment should be individualized, considering specific risk of thrombosis and bleeding [14, 15]. Data from the RIETE registry indicate that the annual recurrence incidence of VTE after discontinuing anticoagulation is approximately 1.5% [16]. It is uncertain whether extending anticoagulation therapy beyond 3 months provides substantial benefits in terms of thrombotic recurrence and bleeding. This is particularly true in patients with cancer, who carry a high risk of major bleeding (MB) (2.3% in the first 3 months of anticoagulant treatment for CRT) [17]. Nonetheless, a recent systematic review found that, during a median follow-up of 13 months, recurrent VTE, MB, and clinically relevant non‐major bleeding (CRNMB) occurred in 3%, 3%, and 4% of patients, respectively, regardless of the presence or the absence of cancer [18]. It is also uncertain whether DOACs at reduced dose may be used efficaciously and safely to reduce the risk of thrombotic recurrence in these patients.

These findings emphasize the critical need for individualized anticoagulation management in these patients [19]. We conducted a retrospective cohort study in women with cancer and CRT-UEDVT, to assess effectiveness and safety of anticoagulation in relation to the duration of treatment.

## Methods

We conducted a retrospective cohort study through a systematic analysis of the electronic database of the Thrombosis Clinic of the Fondazione Policlinico Universitario Agostino Gemelli IRCCS (Rome, Italy). The search was restricted to the period from November 1, 2022, to January 31, 2025. The study was approved by the Institutional Review Board, with protocol number 49,904/18. Patients were included in the analysis in presence of the following criteria: (i) female gender; (ii) age ≥ 18 years; (iii) active cancer, according to the definition of the International Society on Thrombosis and Haemostasis (ISTH) [20, 21]; (iv) confirmed diagnosis of CRT-UEDVT, documented through venous Doppler ultrasound; (v) documented treatment with an anticoagulant (LMWH, fondaparinux, DOAC) at the proper therapeutic dose for at least 3 months; (vi) full access to the follow-up data regarding the primary outcomes of interest of the study. Informed consent was waived due to the retrospective nature of the analysis.

The primary effectiveness outcome of interest was VTE recurrence. The primary safety outcomes of interest were (1) MB and (2) CRNMB, classified according to the definitions provided by the ISTH [22]. Quantification of outcomes was carried out at three different time-points: within the first 3 months of anticoagulant therapy upon diagnosis, between 3 and 6 months of anticoagulant therapy, and between 6 and 12 months of anticoagulant therapy.

A secondary effectiveness outcome was recanalization of the index thrombosis. To assess this outcome, we retrospectively evaluated the records of the colour Doppler ultrasounds performed upon completion of the first 3 months of anticoagulant therapy, after 6 months of anticoagulant therapy, and after 12 months of anticoagulant therapy. In accordance with the existing literature, the recanalization of thrombosis was categorized as absent if the thrombus was unchanged compared to baseline, partial in case of residual thrombus greater than 2 mm in width, and complete in case of residual thrombus of less than 2 mm in width [23].

Discrete variables were summarized with absolute frequencies and percentages. Continuous variables with a normal distribution were summarized with mean and standard deviation, and those with a non-normal distribution with median and interquartile range (IQR). Recurrence and bleeding rate were expressed as rates per 100 person-years. The statistical analysis was performed using STATA SE18^®^.

## Results

We identified 113 women who had the required inclusion criteria and full access to follow-up data upon completion of at least 3 months of anticoagulant therapy. Follow-up data upon completion of 6 months of anticoagulant therapy were available in 106 of these 113 patients (93.8%). Follow-up data upon completion of 12 months of anticoagulant therapy were available in 97 patients (85.8%). The median follow-up was 568.5 days (IQR 300–910). Age at the time of CRT-UEDVT diagnosis was of 56.8 ± 13.3 years. Patients were affected by different cancer type, as follows: ovarian 38.0% (*n* = 43), breast 33.6% (*n* = 38), endometrial 17.7% (*n* = 20), cervical 5.3% (*n* = 6), colon-rectal 4.4% (*n* = 5). A high proportion of patient was affected by a metastatic disease (*n* = 92, 81.4%), with 7.1% patients (*n* = 8) who had brain metastases. Patients carried a PICC in 19.5% (*n* = 22), a chest port in 46.0% (*n* = 52), and an arm port in 34.5% (*n* = 39) of cases. CRT-UEDVT was associated with Pulmonary Embolism (PE) in 9.7% of patients (*n* = 11). Table 1 illustrates the demographic and clinical characteristics of the study population.

**Table 1:** Baseline characteristics of the study population

Age, years, mean ± SD^a^	56.8 ± 13.3
Weight, kg, median (IQR^b^)	63 (56–75)
eGFR^c^ acc. Cockcroft-Gault, mL/min/1,73m^2^, median (IQR)	88 (64.5–115)
*Cancer site*	
Breast, n (%)	38 (33.6)
Ovary, n (%)	43 (38.0)
Endometrium, n (%)	20 (17.7)
Cervix, n (%)	6 (5.3)
Colon-rectum, n (%)	5 (4.4)
Lung, n (%)	1 (0.9)
Bladder, n (%)	1 (0.9)
Other, n (%)	1 (0.9)
Synchronous, n (%)	2 (1.8)
Metastatic cancer, n (%)	92 (81.4)
Intracranial metastases, n (%)	8 (7.1)
*Central Venous Catheter*	
PICC^d^, n (%)	22 (19.5)
Chest port, n (%)	52 (46.0)
Arm port, n (%)	39 (34.5)
Previous VTE^e^, n (%)	13 (11.5)
Previous SVT^f^, n (%)	3 (2.7)
Atrial fibrillation, n (%)	4 (3.5)

^a^SD, Standard Deviation; ^b^IQR, Interquartile Range; ^c^eGFR, estimated Glomerular Filtration Rate; ^d^PICC, Peripherally-Inserted Central Catheter; ^e^VTE, Venous Thromboembolism; ^f^ SVT, Superficial Vein Thrombosis

**Fig. 1:**
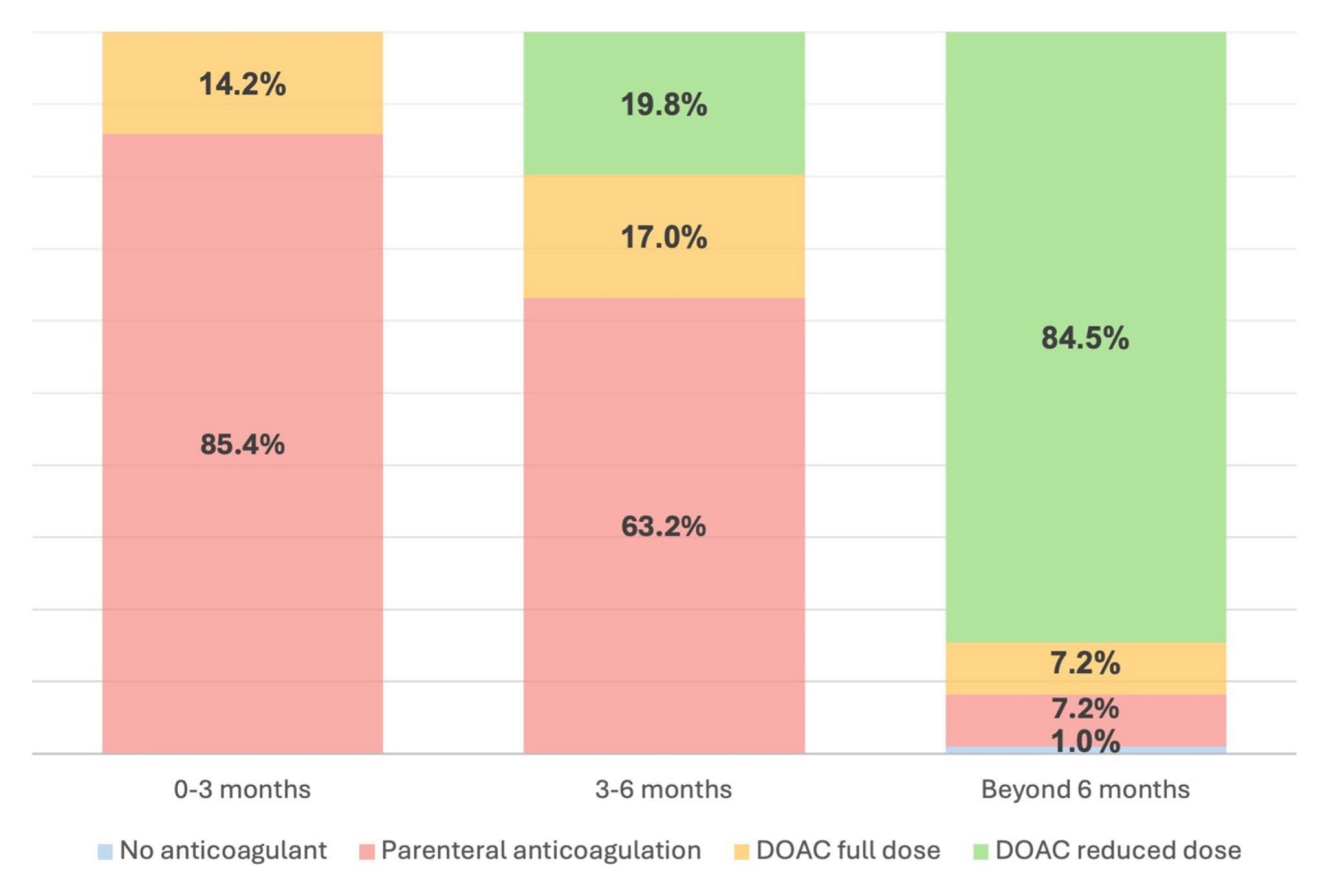
Anticoagulation regimens in the study population at 3, 6 and beyond 6 months. DOAC, Direct Oral Anticoagulant

Figure 1 illustrates the anticoagulation regimens adopted in the first 3 months, between 3 and 6 months, and between 6 and 12 months. In the first 3 months, most patients were treated with parenteral anticoagulation (*n* = 97/113, 85.8%), while a smaller number was treated with DOACs (*n* = 16/113, 14.2%). Between 3 and 6 months, the proportion of patients switched to DOACs raised, with 17.0% (*n* = 18/106) treated with full and 19.8% (*n* = 21/106) with low dose DOACs. Beyond 6 months, a large majority of patients (*n* = 82/97, 84.5%) were treated with low dose DOACs.

In the first 3 months of follow-up, no VTE recurrences were observed. Between 3 and 6 months, 2 patients experienced VTE recurrence, with an incidence rate of 1.9%. Between 6 and 12 months, 2 VTE recurrences were documented, with an incidence rate of 2.1%. The total VTE recurrence rate was of 0.5 events/100 person-years (Table 2). Thrombotic recurrences occurred in patients carrying an arm port, a chest port and a PICC, in 2, 1 and 1 cases, respectively. However, the limited number of events did not allow for an analysis of the results by catheter type.

Regarding bleeding, 4 CRNMBs occurred in the first 3 months (incidence rate 2.7%). Between 3 and 6 months, 1 MB (incidence rate 0.9%) and 3 CRNMB (incidence 2.8%) were documented. Between 6 and 12 months, 1 MB (incidence rate 1.0%) and 5 CRNMB (incidence rate 5.2%) were registered. The total bleeding (MB + CRNMB) rate was of 1.9 events/100 person-years (Table 3).

**Fig. 2:**
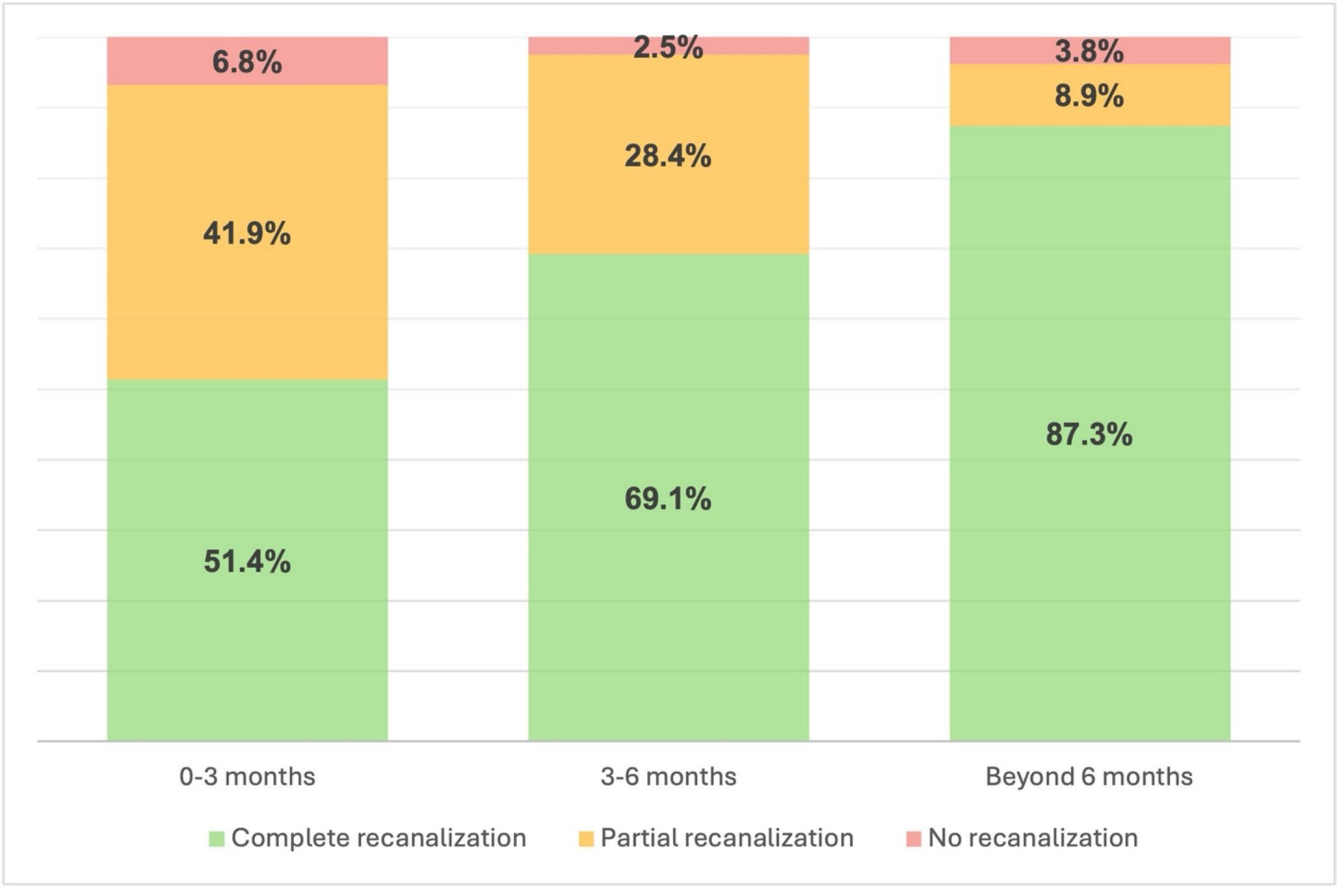
Thrombosis recanalization in the study population assessed by Doppler ultrasound at 3, 6 and beyond 6 months

Regarding recanalization of the index thrombotic event, Doppler ultrasound data were available in 74 out of 113 patients at 3 months, 81 out of 106 patients at 6 months, and 79 out of 97 patients at 12 months. Complete recanalization was observed in 51.4%, 69.1%, and 87.3% of patients with Doppler ultrasound data available at 3, 6, and 12 months, respectively. Figure 2 shows thrombosis recanalization proportion in the study population at different follow-up intervals.

## Discussion

Optimal duration of anticoagulant treatment in patients with CRT-UEDVT is unclear. As mentioned above, there are data from a systematic review showing that 3% of patients have recurrent VTE during a median follow-up of 13 months [18]. Other data from the RIETE registry indicate that the annual recurrence incidence of thrombosis after discontinuing anticoagulation is approximately 1.5% [16]. It is usually recommended to continue anticoagulation until the catheter is in place [24], and patients with cancer, who are the majority of those with CRT-UEDVT, should be kept on anticoagulant therapy until the cancer remains active and/or cancer treatment is ongoing [13]. The result is that many patients with CRT-UEDVT are kept on anticoagulation not just for the treatment of the thrombosis itself, but because the CVC is still in place and/or they have active cancer. The net clinical benefit of such extended anticoagulant regimens is unknown. It is also unknown whether DOACs, especially those at reduced dose, may be effectively and safely used in these patients since the studies on reduced-dose DOACs for extended treatment have not included patients with CRT and UEDVT [25–27]. In the present study, we assessed effectiveness and safety outcomes associated with different lengths of anticoagulation, focusing on women with active cancer and CRT-UEDVT. We decided to focus on women because they are often underrepresented in thrombosis research, particularly in randomized controlled trials and registries [28].

**Table 2:** VTE recurrence

Follow-up Period	Patients, *n*	VTE^a^ recurrence, *n* (%)
0–3 months	113	0
3–6 months	106	2 (1.9)
6–12 months	97	2 (2.1)
Total	113	4 (3.5)

^a^VTE, Venous Thromboembolism

**Table 3:** Bleeding events

Follow-up Period	Patients, *n*	MB ^a^, *n* (%)	CRNMB ^b^, *n* (%)	Total bleeding (MB + CRNMB)
0–3 months	113	0	4 (2.7)	4 (2.7)
3–6 months	106	1 (0.9)	3 (2.8)	4 (3.8)
6–12 months	97	1 (1.0)	5 (5.2)	6 (6.2)
Total	113	2 (1.8)	12 (10.6)	14 (12.4)

^a^MB, Major bleeding; ^b^CRNMB, Clinically Relevant Non Major Bleeding

Regarding the management of CRT-UEDVT, we found that most patients received parenteral anticoagulation during the first three months, with a smaller proportion receiving DOACs. The use of parenteral anticoagulation in the first months is consistent with current guidelines for cancer-associated VTE, where LMWH is often the first-line therapy [13, 29]. However, the use of DOACs in a smaller subset of patients reflects a growing interest in their role in cancer-related thrombosis, though more research is needed to determine their safety and efficacy compared to traditional parenteral therapies in this clinical entity [30].

Regarding VTE recurrence, its annual rate in our study was 0.5%, which is lower than previously reported in the literature among patients with UEDVT [18, 19]. It is also lower than the rate of recurrence documented in patients who are on extended anticoagulation because they had a DVT in a usual site (proximal deep veins of the legs and/or pulmonary embolism) [19, 31–33]. These findings suggest that extended anticoagulation protects cancer patients with CRT-UEDVT from thrombotic recurrences in the long term and is consistent with the notion that extended anticoagulation significantly reduces the risk of VTE recurrence in cancer patients [34]. It is interesting to note that these results were obtained in patients who were mainly treated with DOACs at reduced doses (84.5% of patients) in the time lapse 6–12 months.

Regarding MB and CRNMB, their annual rate in our study was 1.9%, which is higher than the annual incidence of VTE recurrences. This finding indicates that the potential beneficial effects of extended anticoagulation in terms of effectiveness are not free from haemorrhagic events in women with cancer, even if they are treated with low doses of anticoagulants. Our data are consistent with notion that bleeding remains a relevant concern in patients with cancer, particularly as they prolong anticoagulation over an extended period [35, 36]. Among the results of our study, it is interesting to note that anticoagulant treatment beyond 3 months was associated with a greater extent of complete recanalization of the index thrombotic event. This finding suggests that extended anticoagulation may have beneficial effects on this specific effectiveness outcome.

A strength of this study is the notably long median follow-up duration of 568.5 days, which allowed for the evaluation of clinical outcomes in the long term. The focus on female patients is a strength, since women are often underrepresented in clinical trials and observational studies and this can lead to lack of understanding of sex-specific differences in thrombotic and haemorrhagic risks, as well as effectiveness and safety of antithrombotic treatments in women. Moreover, the heterogeneity of cancer sites offers a representative overview of the female oncology population.

This study has some limitations that should be acknowledged. Its retrospective, observational, and monocentric design inherently limits the generalizability of the findings and may introduce potential biases. The allocation of patients to different anticoagulation durations was unbalanced, with most participants receiving long-term therapy. Similarly, different classes of anticoagulants were used, and frequent treatment modifications occurred during the follow-up period. This heterogeneity complicates the interpretation of outcomes and may act as a confounding factor. In addition, venous Doppler ultrasonography was performed in a relatively limited number of patients, which may restrict the accuracy of the assessment. Finally, the inclusion of male patients could have increased the sample size and enabled the investigation of potential sex-related differences with greater statistical significance.

Nevertheless, our study thus provides unique insights into the way women with cancer and CRT-UEDVT are therapeutically managed and how their outcomes are.

In conclusion, the findings of our retrospective cohort study suggest that, in female patients with cancer and catheter-associated thrombosis, a 3-month course of anticoagulant therapy may be sufficient to achieve thrombotic recanalization, while maintaining an acceptable safety profile. Given the low incidence of thromboembolic recurrence and the non-negligible risk of bleeding, the decision to extend anticoagulant therapy beyond this period and its dose warrants careful, individualized consideration. Finally, DOACs appear to be a valuable therapeutic option in this patient population.

## Supplementary Information

Below is the link to the electronic supplementary material.

Supplementary Material 1

## Data Availability

No datasets were generated or analysed during the current study.
