# Supplementary material for: Long term anticoagulation for Catheter-Related deep vein thrombosis of the upper extremities in women with cancer: retrospective analysis of effectiveness and safety outcomes
Source: J Thromb Thrombolysis. 2025 Sep 26;58(8):1127–34. doi: 10.1007/s11239-025-03182-3 (PMC12740948; doi:10.1007/s11239-025-03182-3)
Supplement: Supplementary file 1 — Supplementary Material 1 [file 11239_2025_3182_MOESM1_ESM.pdf]

Study Cohort

113 women with **active cancer** who had received anticoagulant treatment for Catheter-related thrombosis

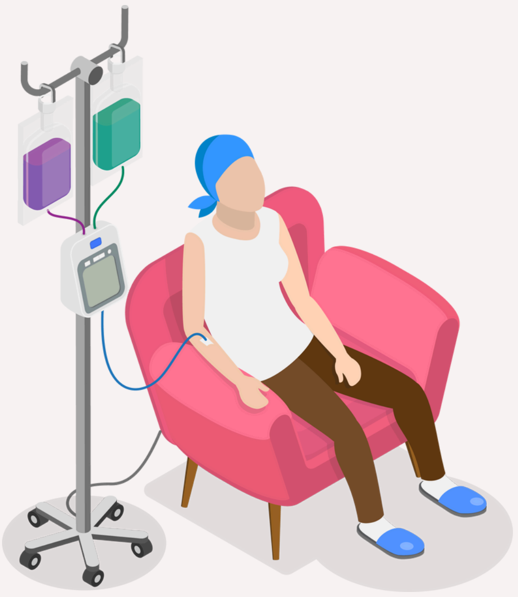

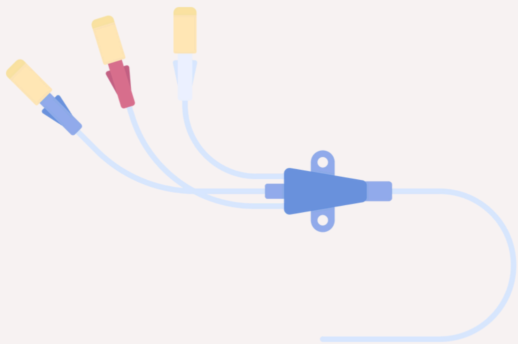

Catheter-related thrombosis (CRT): start anticoagulation.

Clinical and ultrasound follow-up with monitoring of events related to **anticoagulant therapy**.

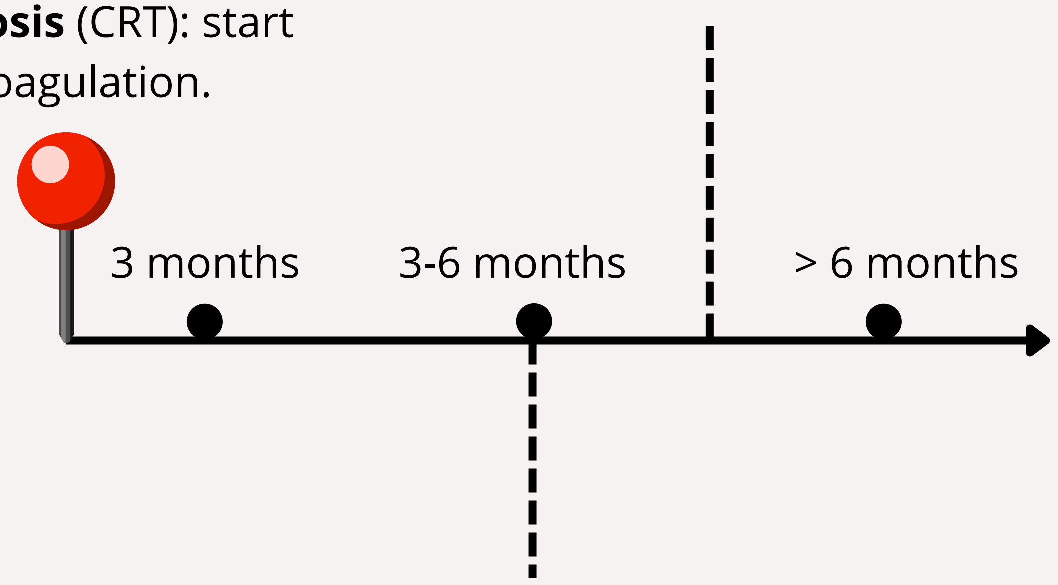

Median follow-up 568.5 days

Outcomes

Recurrence of venous thromboembolism

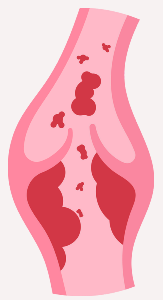

Recurrence rate of venous thomboembolism was 0.5 events/100 person-years

Bleeding events

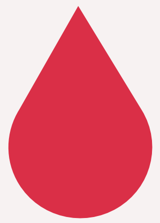

The total relevant bleeding rate was of 1.9 events/100 person-years

Thrombosis recanalization

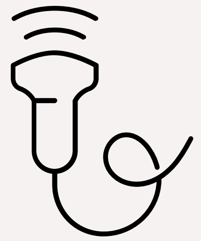

The rate of thrombosis recanalization after six months was 87.3%
